# Minimal symbol grounding through language statistics: An information theoretic approach quantifying uncertainty

**DOI:** 10.3758/s13423-026-02995-4

**Published:** 2026-08-31

**Authors:** Max M. Louwerse

**Affiliations:** Department of Computational Cognitive Science, Tilburg University, Warandelaan 2, Tilburg, 5037 AB The Netherlands

**Keywords:** Language, Symbol grounding, Information theory, Language statistics, Embodied cognition

## Abstract

One of the fundamental issues in the cognitive sciences concerns the question of how language attains meaning. The current study investigated how the extent to which language statistics and symbol grounding reduce uncertainty about word meaning can be quantified. Using Shannon’s information theory, conditional entropy of the valence of a word was quantified without grounding (baseline), with minimal symbol grounding and with full grounding. By using a mini lexicon as a principled illustration, later extended to a full lexicon, the entropy of phonological cues, frequency cues, and word embeddings were compared to a symbol-grounding case when predicting word valence. Phonology and frequency provided small but measurable reductions in uncertainty, which word embeddings propagated through a lexical network, particularly once only a few seed words were grounded. The results show that minimal grounding, combined with language-statistical structure, can substantially reduce uncertainty about word valence. The evidence of minimal symbol grounding sheds light on the symbol-grounding problem and encourages new and quantifiable perspectives on past and present theories on language and cognition.

## Introduction

The question of how language attains meaning has played a central role in the cognitive sciences. Today the question is even more prominent because of the increased interest in large language models (LLMs) and the question about whether these models realize meaning (Harnad, 2024; Piantadosi & Hill, 2022). Answers regarding how language attains meaning can ultimately be traced back to the question about whether the linguistic sign is arbitrarily linked to its meaning, such that a word does not reveal any of the meaning it conveys. This position was most notably formalized by Saussure (1916). Hockett repeated the arbitrariness principle as one of the fundamental design features of language: “Human language […] is almost entirely arbitrary in its semantic conventions” (Hockett, 1958; p. 577). The arbitrariness assumption creates a symbol-grounding problem (Glenberg, 2026; Harnad, 1990), the solution of which lies in grounding language in perceptual and sensorimotor experiences (Barsalou, 1999; Glenberg, 1997). The necessity to ground symbols is illustrated in the Chinese room thought experiment (Searle, 1980): If a person in a room not knowing Chinese receives arbitrary Chinese symbols and translates these into other arbitrary Chinese symbols using a rule ledger, anybody would conclude the person in the room does not understand Chinese. Instead, the person translates symbols into other symbols, creating a symbol merry-go-round (Harnad, 2024). Only when arbitrary (Chinese) symbols are grounded to their referents, i.e., entities outside the language system, do they become meaningful. A similar point has been made more recently when the rule ledger became an eavesdropper who induces patterns by overhearing lots of language-in-use that appears to be fluent yet lacks understanding because of the absence of grounding (Bender & Koller, 2020).

If linguistic symbols must always be grounded to be meaningful, this poses major challenges to fundamentally non-grounded LLMs. Consequently, the mechanisms LLMs use ought to be considered fundamentally different from the way humans assign meaning to language. LLMs might be a useful tool to summarize text, i.e., they might be useful eavesdroppers (Bender & Koller, 2020), but they cannot in any way explain how language attains meaning, because they face the symbol-grounding problem (Pavlick, 2023).

Even though a strong arbitrariness assumption still motivates some versions of the grounding debate (Glenberg, 2026), many researchers agree that the relation between linguistic form and meaning is neither wholly arbitrary nor wholly motivated. Indeed, there is an increasing amount of evidence that language exhibits a balance between arbitrary and non-arbitrary structure, the latter including iconicity and systematicity (Blasi et al., 2016; Dingemanse et al., 2015; Monaghan et al., 2014), even showing that language systematically encodes perceptual information (Louwerse, 2010, 2018, 2021). Evolutionary processes have thus shaped the language system into a convenient tool (Christiansen & Chater, 2008, 2022), allowing language users to utilize this tool, at least for quick, good-enough representations (Ferreira et al., 2002). Complementary to these encodings, language users can ground linguistic symbols (Barsalou, 1999).

The debate on symbol grounding has primarily focused on whether or not language must be grounded (De Vega et al., 2012), even though terminology may have obfuscated the discussion (Reilly et al., 2025). One explanation the question has been posed in a binary way is that evidence in favor of symbol grounding often comes from experiments that typically look at differences rather than relative contributions (Louwerse et al., 2015), with evidence for one (perceptual) condition being evidence against the other (language statistics) condition (Borghi et al., 2004; Connell & Lynott, 2012; Glenberg & Kaschak, 2002; Glenberg & Robertson, 2000; Pecher et al., 2003; Zwaan & Yaxley, 2003; Zwaan et al., 2002, 2004).

Another explanation for why the question of to what extent grounding is needed for language to become meaningful has not been addressed is that a formal comparison tailored to grounding and language-statistical cues has been lacking. A measure that formalizes uncertainty about the meaning of a word allowing for a comparison across language statistical cues and grounding can be found in information theory. Shannon’s information theory allows for quantifying information so that the limits of communication can be measured with the goal of optimizing representation and delivery of symbols (Shannon, 1948). Information theory has been used to demonstrate how efficiency has shaped human language (Gibson et al., 2019).

The present study asked first what the information uncertainty is in a baseline condition whereby the language system is fully arbitrary, then in a condition with language statistical structures, and finally in a condition where a number of words are grounded and the remaining words are connected by language-statistical cues. To answer these questions the semantic dimension of valence is used here as a tractable example of meaning because large normed datasets are available and because affective meaning is relevant to debates about grounding in bodily, experiential, and linguistic sources of semantic knowledge. The analysis does not at all assume that valence exhausts word meaning, nor that effects observed for valence automatically generalize to all semantic dimensions. Instead, valence is used here as a transparent test case for quantifying how much information language-statistical cues reduce uncertainty about one semantic dimension.

## Methods

The current study is a principled illustration of how much uncertainty about the semantic dimension of valence can be reduced with language statistics and direct grounding. Because it is a principled illustration, a mini lexicon of 30 words was first used, and variables (e.g., valence, phonology, frequency, and word embeddings) were defined where possible in a dichotomized way to maximize insight. A second study used an entire list of 13,915 English valence norm lemmas. For the mini lexicon, 15 words with the highest positive and negative valence scores were selected from the Warriner et al. (2013) list (Table 1). For the full lexicon, valence was dichotomized using a median split of the valence scores (Warriner et al., 2013).

**Table 1:** Thirty-word mini-lexicon with valence scores, valence labels, and three kinds of language-internal cues: the nasal onset and word frequency

Word	Valence score (Warriner et al., 2013)	Valence	Nasal onset	Log frequency	cos (*positive*)	cos (*negative*)
vacation	8.53	positive	0	17,068	.085	.062
happiness	8.48	positive	0	15,532	.286	.186
happy	8.47	positive	0	17,454	.388	.214
enjoyment	8.37	positive	0	15,070	.158	.106
Christmas	8.37	positive	0	16,081	.051	.022
fun	8.37	positive	0	18,162	.248	.145
fantastic	8.36	positive	0	16,189	.272	.138
lovable	8.26	positive	0	13,105	.197	.137
free	8.25	positive	0	20,096	.176	.157
hug	8.23	positive	0	14,437	.17	.103
magical	8.23	positive	0	15,115	.196	.153
delight	8.21	positive	0	15,192	.122	.048
joy	8.21	positive	0	16,016	.232	.119
joyful	8.21	positive	0	13,630	.424	.246
relaxing	8.19	positive	0	15,193	.239	.152
comatose	2	negative	0	12,050	.053	.034
homicidal	2	negative	0	12,045	.092	.159
poverty	2	negative	0	16,441	.056	.107
sabotage	2	negative	0	13,462	.12	.175
infection	2	negative	0	16,339	.171	.204
fatal	2	negative	0	15,270	.127	.193
slavery	2	negative	0	14,911	.039	.122
mutilate	2	negative	1	11,278	-.031	.083
foreclose	2	negative	0	12,013	.044	.125
excrement	2	negative	0	12,059	.038	.122
mistrust	2	negative	1	12,882	.19	.273
pollution	2	negative	0	16,043	.096	.211
maggot	2	negative	1	11,779	.056	.107
pain	2	negative	0	17,267	.141	.203
alcoholism	2	negative	0	13,830	.07	.145

### Language statistics

#### Phonological features

To demonstrate the effect of sound symbolism on determining the meaning of a word in the mini lexicon, phonological properties have been reported to carry weak but systematic semantic biases in valence (Louwerse & Qu, 2017). Specifically, a nasal onset of the word increases the likelihood that a word has a negative connotation with a .55 probability in English, a finding just above chance but reliable (Louwerse & Qu, 2017). Nasal onset in the current analyses was computed using the CMU Pronouncing Dictionary. Each word was looked up in CMUdict (Lenzo) to obtain its ARPAbet pronunciation.

#### Word frequency

Word order has also been shown to predict valence, presumably because language has a positivity bias (Dodds et al., 2015). That is, positive words more often precede negative words in a 5-gram window (Benor & Levy, 2006; Hutchinson & Louwerse, 2013). Moreover, there are more positive words than negative words, and we know that frequent words precede infrequent words (Lehrer, 1985), yielding the hypothesis that the frequency of the word is predictive of its valence. Word frequencies were obtained from Google Web 1 T corpus (Brants & Franz, 2006). Frequency as a predictive cue was operationalized as a binary cue by applying a median split to log10-transformed frequency values. Words above the median were coded as high frequency and words at or below the median as low frequency.

#### Word embeddings

Distributional semantics, or word embeddings, assume that a word’s meaning can be inferred from the linguistic contexts it appears in (Firth, 1957). Each word acts as a point (a vector) in a continuous, high-dimensional space learned from large text corpora, such that words used in similar contexts end up with similar vectors (Landauer & Dumais, 1997; Mikolov et al., 2013).

Here 300 dimensional fastText word vectors were used, created from Wikipedia and Common Crawl data (Grave et al., 2018). Specifically, the word-embedding method was the same as the one used in Lingualyzer (Linders & Louwerse, 2024). During training, the model adjusted the word vectors such that words that reliably predict similar contexts have vectors that are close together. After training, semantic similarity was computed using cosine similarity between vectors. To dichotomize the word embeddings, the cosine value for each word of the lexicon was computed with two words with extreme positive and negative valence scores, *positive* and *negative*. A word was classified as positive if its cosine similarity to the word *positive* was greater than its cosine similarity to the word *negative*, otherwise it was classified as distributionally negative.

## Results

### Mini lexicon

#### Baseline

Information theory was applied here first to a mini lexicon. If language statistics were to not at all predict valence, merely hearing the word gives no information about its valence, and the valence is maximally uncertain (*p* = .﻿5). Using Shannon’s entropy function H(V), the entropy of Valence, with the output variable being v ∈ {positive, negative}, entropy translates to 1 bit.

1 $$H(V)=-\sum\nolimits_{v}p(v)\;{\mathrm{log}}_{2}\;p(v)$$

If, on the other hand, class probability deviates from .5 due to systematicity in language, entropy is reduced to < 1 bit by biasing whether a word is positive or negative.

#### Phonological features

When using phonological features to the 30-word mini lexicon by computing the nasal onset of a word, three words that start with a nasal are negative. Overall classification accuracy if nasal predicts negative and non-nasal predicts positive valence was (18/30) or .60, notably an extremely noisy probability given the extremely small dataset. After observing whether a word begins with a nasal consonant, uncertainty is reduced to the conditional entropy computed by Eq. 2.

2 $$H\left(Y\left|X\right.\right)=-\sum\nolimits_{x}P\left(x\right)\sum\nolimits_{y}P\left(y\left|x\right.\right){\mathrm{log}}_{2}\;P\left(y\left|x\right.\right)$$

Conditional entropy $H\left(Y\left|X\right.\right)$ is the average uncertainty remaining about Y after observing X. That is, for each value x, the entropy of Y is computed under $P\left(y\left|x\right.\right)$, weighted by how likely x is. With all three nasal words being negative, no uncertainty exists about the nasal words. For the non-nasal words, 12/27 = .444 for the negative words and 15/27 = .556 for the positive words. Consequently,

$$H\left(Y\left|X\right.\right)=-\left(0.444\;{\mathrm{log}}_{2}\left(0.444\right)+0.556\;{\mathrm{log}}_{2}\left(0.556\right)\right)\approx 0.991$$

Conditional entropy averages the two groups by their sizes:

$$H\left(Y\left|X\right.\right)=(3/30)(0)+(27/30)(.991)\approx .892$$

Consequently, in information theory mutual information can be computed using

3 $$I\left(X;Y\right)=H\left(Y\right)-H\left(Y\left|X\right.\right)$$

That is, 1-.892=.108. This means that a reduction of 10.8% of the baseline uncertainty is obtained using a very simple phonological feature such as a nasal-first rule.

#### Frequency

When considering the frequency of a word, positive valence words tend to be more frequent compared to negative valence words. When considering a median split on the log frequency of the words of the mini lexicon, 10/15 low-frequency words are negative (.667), 5/15 are positive (.333) yielding:

$$H \left(Y \right| \text{low frequency})= -\left(.667\;{\mathrm{log}}_{2}\;(.667)+.333\;{\mathrm{log}}_{2}\;(.333)\right)$$

For the high-frequency words 5/15 are negative and 10/15 are positive:

$$H \left(Y \right| \text{high frequency})= -\left(.333\;{\mathrm{log}}_{2}\;(.333)+.667\;{\mathrm{log}}_{2}\;(.667)\right)$$

Consequently, the conditional entropy was 0.918 bits, and the mutual information was 1 ˗.918 = 0.082 bits. Knowing whether a word is frequent removes about 8.17% of uncertainty about valence compared to a baseline that assumes a 50% chance.

#### Word embeddings

Of the 14 words whose highest cosine match was with *negative*, all 14 were truly negative. Of the 16 words whose highest cosine match was with *positive*, 15 were truly positive and 1 was truly negative (*comatose*). Consequently,

$$\begin{array}{l}H(Y\mid\text{positive cosine match})= \\-\left(0.938\log_2(0.938)+0.063\log_2(0.063)\right)\approx0.337\text{ bits}.\end{array}$$

whereas *H*(*Y* | *X* = negative cosine match) = 0 bits. Weighted by group:

$$H\left(Y\left|X\right.\right)=\left(14/30\right)\left(0\right)+\left(16/30\right)\left(.337\right)\approx .180\;\mathrm{bits}.$$

The mutual information is:

$$I\left(Y;X\right)=1- .180=.820\;\mathrm{bits}.$$

Mutual information quantifies the absolute reduction in uncertainty about an outcome obtained. To express this reduction relative to the baseline uncertainty, the mutual information can be normalized using Eq. 4:

4 $$U\left(Y\left|X\right.\right)=\frac{I\left(Y;X\right)}{H\left(Y\right)}=1-\frac{H\left(Y\left|X\right.\right)}{H\left(Y\right)}$$

This normalized measure expresses the proportion of the initial uncertainty in (*Y*) that is removed by knowledge of (*X*). Albeit not mathematically equivalent, it is analogous in interpretation to the effect-size measure of *R*^2^ (Chen et al., 2018). In the present study, positive and negative words were balanced, resulting in a baseline entropy of 1 bit. Given the baseline entropy of 1 bit, a mutual information value of 0.820 bits corresponds to a normalized mutual information value of *U(Y | X)* = .820, meaning that 82.0% of the initial uncertainty is removed.

#### Minimal grounding

One may object that these findings are a perfect example of the symbol merry-go-round argument (Glenberg, 2010; Harnad, 1990). The anchor-based analysis shows that words can be classified according to whether they are distributionally closer to the word *positive* or the word *negative*, but it presupposes that these two anchors already identify the positive and negative poles of the embedding space. Word embeddings therefore provided relational structure, but this structure does not by itself explain how its semantic orientation is established. Ultimately, some external grounding or other source of semantic asymmetry is required. The question then becomes how much grounding is needed before language-statistical structure can propagate that information to the remaining words.

To address this question, valence information now was now entered in the model only through the (*k*) words randomly selected as grounded. For each ungrounded word, cosine similarity was computed with every grounded word, and the ungrounded word was assigned the valence of its most similar grounded neighbor. In this way, (*k*) represented the complete amount of grounded valence information available to the model.

For the purposes of this study, imagine a language learner learning new words, each of the words of the mini lexicon. Assume the learner knows that half of the words have a positive valence, the other half have a negative valence. The entropy that started with 1 bit (.5 chance) is reduced with each word being grounded, reaching a minimal uncertainty after 29 words.

Grounding simulations were conducted by randomly sampling grounded words without replacement. Sampling was uniform over words, so the proportion of positive and negative words in each grounded set varied naturally. For each k, grounded words were sampled without replacement. For each sampled split, conditional entropy was recomputed over the remaining ungrounded set ${U}_{k}$, scaled by $\left|{U}_{k}\right|/N$. For each value of *k*,

5 $${H}_{k}\left(C\right)=\frac{\left|{U}_{k}\right|}{N}H\hspace{0.17em}\left({Y}_{{U}_{k}}\left|{C}_{{U}_{k}}\right.\right)$$

where *N* is the total number of words in the lexicon, ${U}_{k}$ is the set of words that remain ungrounded after *k* words have been grounded, ${Y}_{{U}_{k}}$ contains the valence labels of those ungrounded words, and ${C}_{{U}_{k}}$ contains the information supplied by cue condition of those words. The conditional entropy $H\left({Y}_{{U}_{k}}\left|{C}_{{U}_{k}}\right.\right)$ therefore quantifies the uncertainty remaining about the valence of the ungrounded words after the cue has been observed. This uncertainty is multiplied by $\left|{U}_{k}\right|/N$ because the *k* grounded words have known valence and consequently contribute zero uncertainty. Thus, ${H}_{k}\left(C\right)$ expresses the average remaining uncertainty across the complete lexicon, including both grounded and ungrounded words.

At k=0, all words remain ungrounded, so $\left|{U}_{0}\right|/N=1$. The remaining uncertainty therefore corresponds to the baseline entropy or conditional entropy associated with each condition

$$\begin{aligned} &H_o\,(\text{grounding only}) = H(Y \mid \mathrm{grounding}) = 1\,\mathrm{bit}.\\ &H_o\,(\mathrm{nasal}) = H(Y \mid \mathrm{nasal}) = 0.892\,\mathrm{bits}.\\ &H_o\,(\mathrm{frequency}) = H(Y \mid \mathrm{frequency}) = 0.918\,\mathrm{bits}.\\ &H_o\,(\mathrm{embedding}) = H(Y) = 1\,\mathrm{bit}. \end{aligned}$$

Before direct grounding begins, nasal onset and frequency already function as ordinary conditional-entropy cues, whereas word embeddings do not yet help because there is no grounding. The combined cue condition has the lowest value at *k* = 0, *H*_*0*_ (nasal + frequency) = 0.851 bits, because it uses both nasal onset and frequency. At *k* = 2, a total of 28 words remain ungrounded, so every conditional entropy is multiplied by 28/30 = .933. If the cue-specific conditional entropy among the ungrounded words was approximately equal to its value at k=0, then:

$${H}_{2}\left(\mathrm{nasal}\right)\approx 0.933\times 0.892=0.832\;\mathrm{bits}.$$

$${H}_{2}\left(\mathrm{frequency}\right)\approx 0.933\times 0.918=0.856\;\mathrm{bits}.$$

Note here that the nasal and frequency numbers are approximations, not the actual means across random draws.

For word embeddings, the mean conditional entropy among the ungrounded words across the random draws was .722 bits. Consequently:

$${H}_{2}\left(\mathrm{embedding}\right)=0.933\times 0.722=0.674\;\mathrm{bits}.$$

Note that word embeddings thus become very informative. With two grounded words, the ungrounded words can be closer to one grounded anchor than the other, so embedding space begins to partition the remaining words in a way that moves them to positive or negative valence. The drop in entropy from the relevant baseline can be read as information gained from language statistics. For example, at *k* = 3 the grounding-only uncertainty is 0.898 bits, while the all-cue uncertainty is 0.418 bits. The additional reduction due to the cues at that step is therefore about 0.480 bits beyond direct grounding alone (0.898 ˗ 0.418 = 0.480 bits).

**Fig. 1:**
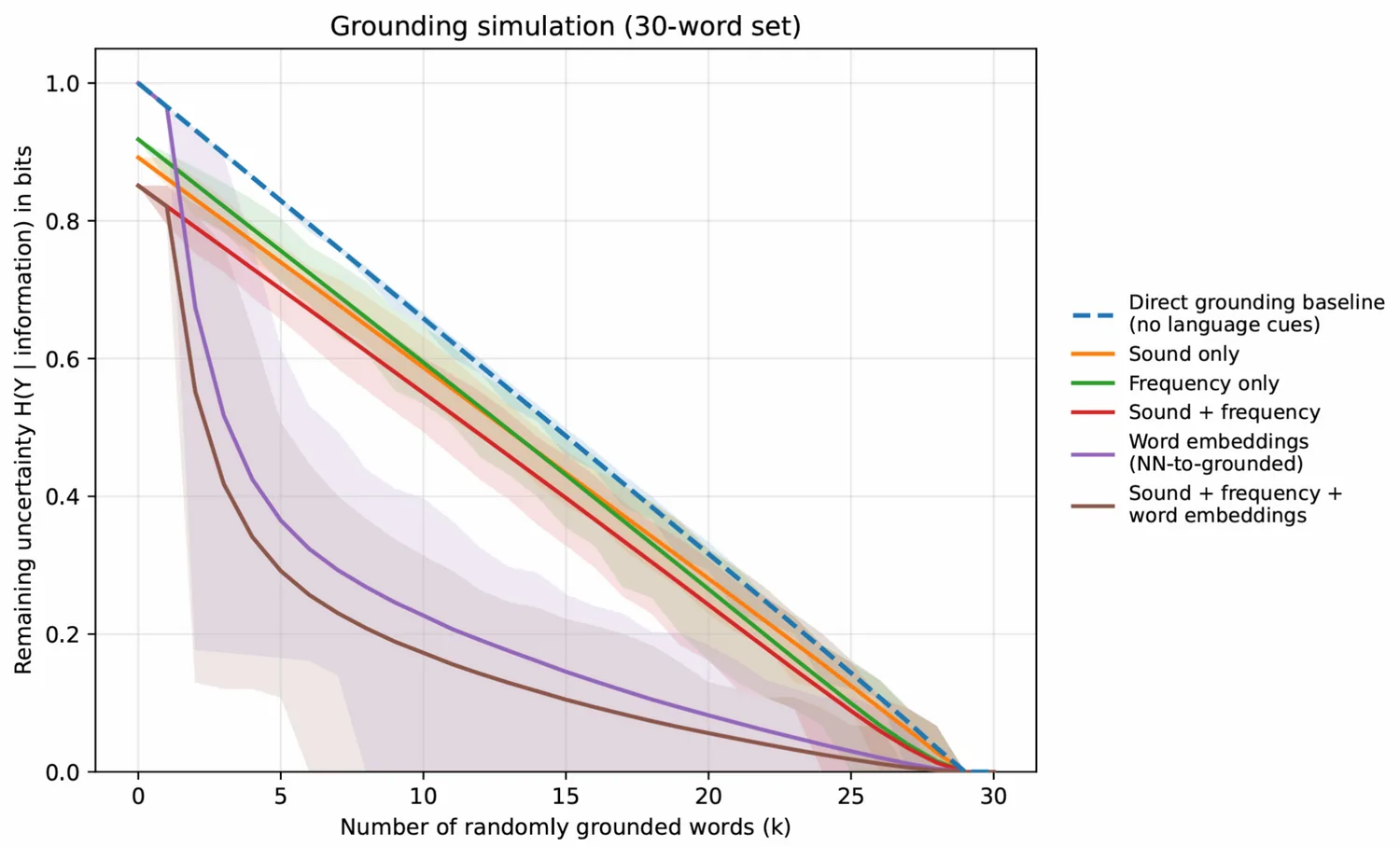
Overview of number of grounded words in the 30-word mini lexicon in relation to the remaining uncertainty for each of the language statistical cues*.* The figure shows the 10th–90th percentile range of remaining uncertainty across random draws (i.e., a variability band showing how much the outcome depends on which specific words happened to be grounded)

Figure 1 shows the remaining uncertainty in bits as a function of the number of grounded words. This quantification is agnostic regarding how hard it is to compute each cue (sound, frequency, word embeddings), how hard it is to acquire grounding (experience, supervision, memory), and how hard it is to apply a rule online. However, language users generally strive to minimize cognitive effort (Ferrer i Cancho & Solé, 2003; Linders & Louwerse, 2023; Zipf, 1949). Without an effort term, the best strategy may be to use all information available that reduces uncertainty most. When cognitive effort is included, the relevant question becomes which curve gives the best reduction per unit cost.

These findings suggest language statistics supply much of the valence-relevant structure, while grounding supplies the few missing bits needed to propagate that structure throughout the language system. The findings also show that minimal grounding is needed to bootstrap meaning across the language statistics given the language statistical patterns. This is backed up by experimental evidence providing evidence that switching verbal (linguistic) and non-verbal (perceptual) codes takes effort (Paivio, 1990). Moreover, when language users need to decide quickly, they are known to rely more on linguistic cues than on non-linguistic cues (and vice versa if they take longer to process information) (Louwerse & Connell, 2011; Louwerse & Jeuniaux, 2010).

A mini lexicon, however, is not representative of the tens of thousands of words an average language user knows. The demonstration nevertheless is very much representative of human language. Not only were the (small number of) words collected from actual language data, but language statistics were also derived and computed from empirical data. Most importantly, whether the lexicon consists of tens of thousands of words does not invalidate the conclusions drawn here. The fact that only extreme valence values have been selected here might however explain the drop in remaining uncertainty preventing a generalization.

### Full lexicon

The quantification of uncertainty using language statistics and grounding serves as an illustration to gain insight into Shannon’s information theory. Useful as a principled illustration, it does not say much about the language system at large. To demonstrate how the findings for the mini lexicon generalize across a large lexicon, the full Warriner valence list of 13,915 lemmas was used. Of these, 107 items were removed because they were multi-word combinations or personal names/proper-name expressions, such as *action figure*, *aircraft carrier*, and *Grand Canyon*. Only words for which the phonology and frequency information was available were selected next. A total of 415 of the 13,808 post-exclusion entries lacked usable CMUdict pronunciation and 606 words lacked a frequency. The full analysis contained 12,787 words, 6,420 with positive and 6,367 with negative valence based on a median split.

Given that the valence class distribution is almost exactly balanced, entropy is 1 bit. In the full lexicon, 1,857 words had a nasal onset. Of these, 989 were negative and 868 were positive, so *P*(negative | nasal onset) = .533. Among the 10,930 non-nasal words, 5,378 are negative and 5,552 are positive, so *P*(negative | non-nasal) = .492. Consequently, conditional entropy was .999 bits and mutual information .001 bits. In the full lexicon, nasal onset thus carried almost no information about valence.

Frequency was again coded using a median split on log frequency. In the low-frequency group, 3,880 of 6,394 words were negative (*P* = .607), whereas in the high-frequency group, 3,906 of 6,393 words were positive (*P* = .611). Consequently, conditional entropy was .965 bits and mutual information .0345 bits. Whereas nasal onset did not reduce uncertainty, frequency provided a modest but clearer signal than nasal onset in the full lexicon, removing about 3.45% of the original one-bit uncertainty.

Word embeddings were again defined by whether a word was closer to the word *positive* or to the word *negative*. Of the 8,062 words oriented toward *negative*, 5,128 were actually negative and 2,934 were positive. Of the 4,725 words oriented toward *positive*, 3,486 were actually positive and 1,239 were negative. Word embeddings yielded a conditional entropy of.903 bits, and mutual information of.097 bits removing about 9.7% of the baseline uncertainty.

**Fig. 2:**
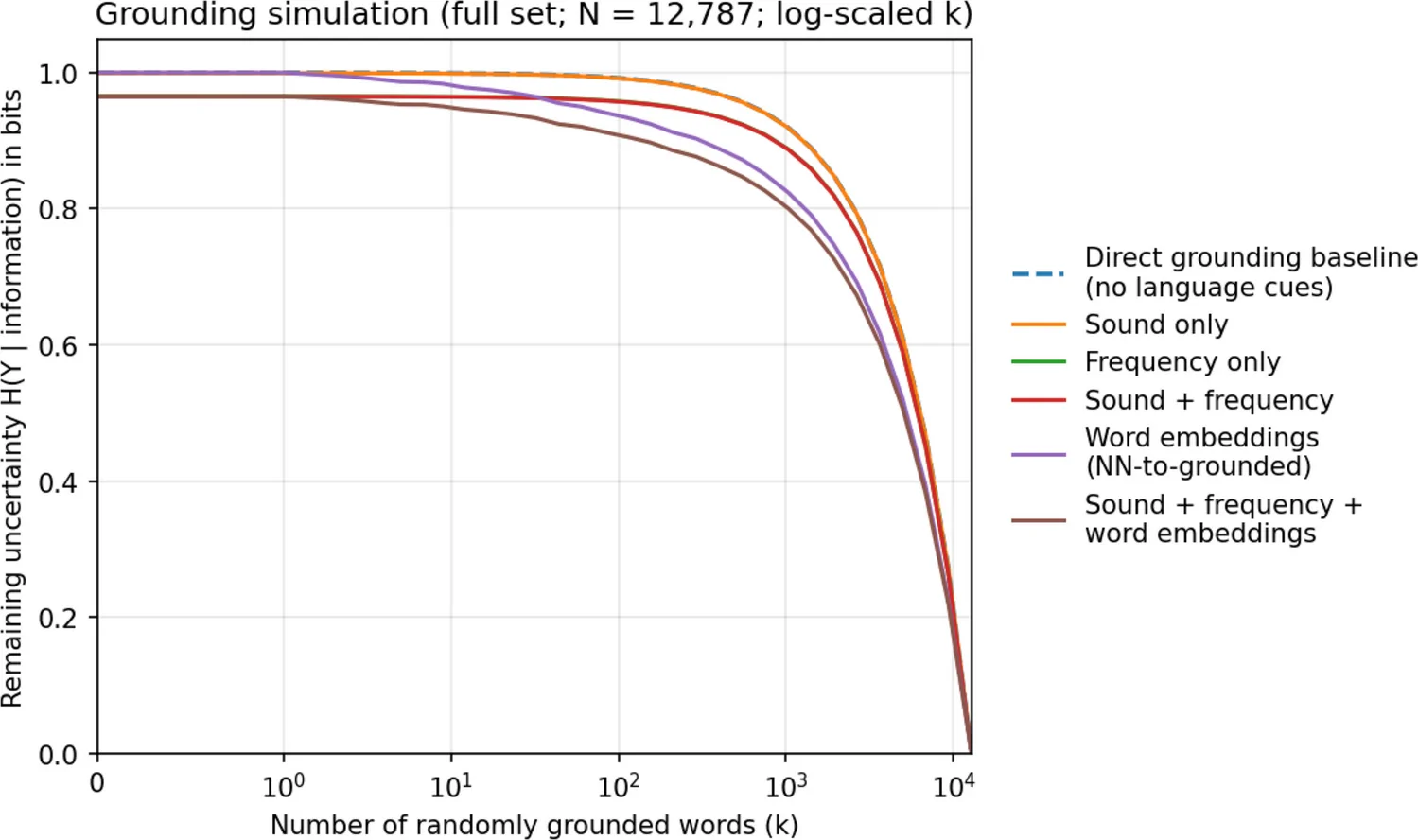
Overview of the number of grounded words in the full lexicon in relation to the remaining uncertainty for each of the language statistical cues*.* The figure shows the 10th–90th percentile range of remaining uncertainty across random draws

**Fig. 3:**
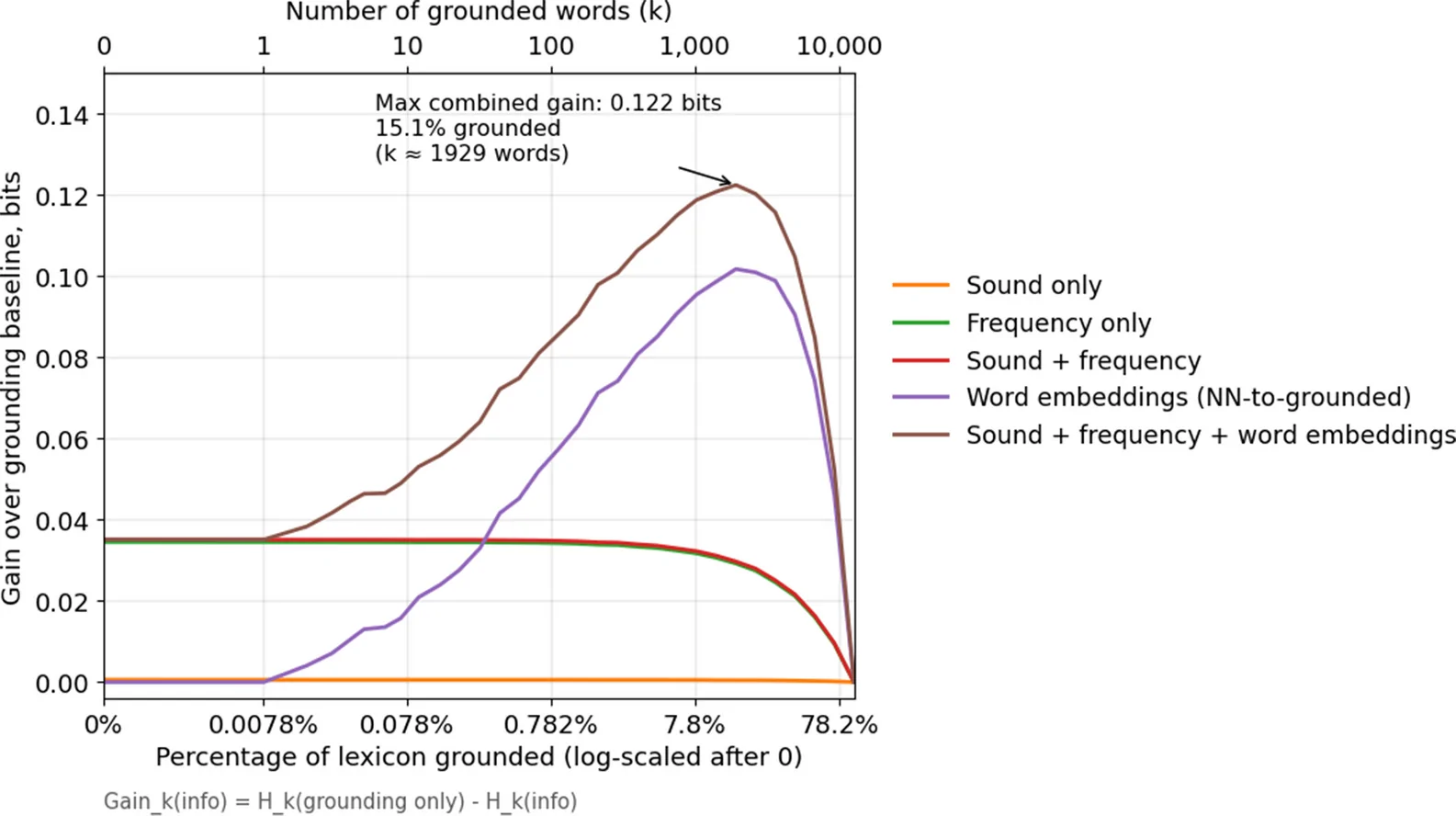
Gain from language statistics over direct grounding (baseline)

When the minimal-grounding exercise for the mini lexicon was repeated for the full lexicon, the same procedure was used: the fixed anchors *positive* and *negative* were omitted, and each ungrounded word was assigned the valence of its most similar grounded word. The results are now less dramatic than in the mini lexicon because the lexicon is much larger with half of the valence values having average scores, *N* = 12,787 words. Figure 2 shows that remaining uncertainty decreases mostly because more words are directly grounded. The language statistics curve lies below the grounding-only baseline, but the separation is modest initially. However, this does not mean language statistics are irrelevant. Figure 3 makes the contribution of language statistics clearer by plotting the extra gain over grounding alone. This gain plot shows that language statistics help most in the intermediate range, not when almost no words are grounded, and not when most words are already grounded, but once enough grounded examples exist for language statistics to generalize to ungrounded words. The largest gain occurs for the combined cue condition, sound + frequency + word embeddings, at approximately 15.1% of the lexicon being grounded.

In line with other studies (Louwerse, 2010, 2018), what Figs. 1–3 show is that grounding and language statistics are best interpreted as complementary: grounding supplies known meanings, while language statistics help propagate that information to ungrounded words.

## Discussion

This study demonstrated a formal comparison of uncertainty under language-internal cues alone, direct grounding alone, and their combination. Findings demonstrated that minimal grounding together with language-internal structure improves uncertainty reduction. A principled illustration was used on a small number of 30 words to explain information theory and for a full lexicon of almost 13,000 words. The results demonstrate quantifying information from the perspective of information theory shows that relatively simple language statistical cues such as phonological, frequency, and word-embedding properties reduce entropy. Minimal grounding already reduces the entropy of the meaning of a word. The findings based on information theory match conclusions drawn from experimental studies, showing that minimal grounding is sufficient for extracting meaning from language (Louwerse, 2018). In a principled illustration, this paper has shown how language statistics – phonology, frequency, and word embeddings – inform the valence of a word in a mini lexicon. Moving beyond this illustration, a full lexicon yielded similar conclusions.

The findings from the mini lexicon were clearest, showing that a small number of grounded words in combination with language statistics already yields a significant drop of entropy after two words are grounded. However, albeit useful as a principled illustration, the findings from the mini lexicon may overstate the role of language statistics and minimal grounding. The full lexicon shows a more moderate effect for language statistics, yet still shows the largest drop in entropy when the language statistical cues are combined with grounding. Given its size, the full lexicon may, on the one hand, not be considered a toy example; however, on the other hand, the majority of the words with a moderate valence score may have underestimated the role of language statistics and grounding.

The focus on (only) the single dimension of valence for which minimal grounding is demonstrated can be considered a major limitation. For demonstration purposes, however, it suffices, as there is considerable evidence that language statistical cues such as phonology, word order, frequency, and word embeddings predict semantic dimensions. For instance, phonological cues have been shown to predict perceptual magnitude and size (Winter & Perlman, 2021), sound, motion, and texture (Dingemanse et al., 2015), as well as semantic categories (Monaghan et al., 2014). Word order has been shown to predict vertical relations (Louwerse, 2008), gender, and authority (Hutchinson & Louwerse, 2018). Word embeddings have been shown to predict social relations (Hutchinson & Louwerse, 2018) and geographical information (Louwerse & Benesh, 2012; Louwerse & Zwaan, 2009), valence (Hollis & Westbury, 2016; Warriner et al., 2013), and modality (Louwerse & Connell, 2011). Language statistics predict a range of semantic dimensions, valence being one of them. More importantly, the findings reported here are not meant as a general solution to the full symbol-grounding problem, but as a test case about how uncertainty reduction can be quantified and consequently how much external grounding is needed when language-internal statistics are available.

Finally, one could raise the concern that only three language statistical cues might not maximize the role of language statistics at large. The point of this paper was not to demonstrate the maximum role of language statistics, but instead to illustrate how to quantify language statistics and grounding. Several studies demonstrate additional language statistical features that may be considered for future research, including length, orthographic neighborhood, concreteness, and morphological structure (Balota et al., 2007; Yarkoni et al., 2008).

It is certainly true that language statistical cues correlate, which may lower their independent effects on entropy. It is also true that extracting meaning requires more than a binary decision – even if the focus is on a single dimension such as valence. However, given that there is a range of correlating linguistic features that may be predictive of a multi-dimensional space of meanings, the demonstration presented in this paper provides a first step in understanding how minimal grounding allows for answering the question of how language attains meaning.

The current findings may trigger a renewed interest in scientific theories of language, and provide renewed motivation to consider other long-held assumptions. If language statistics affect or bias meaning, it revitalizes theories such as linguistic relativity, which asserts that language may shape worldview or cognition (cf. Kemmerer, 2023). Different language statistical cues across languages then presumably bias meaning extracted from these languages differently (Linders & Louwerse, 2024).

Finally, evidence that language encodes patterns that direct meaning extraction sheds light on two cognitive aspects in which humans have been shown to differ from non-human species. One is that humans excel in language processing (Tomasello, 2009), the other is that humans excel in finding patterns in data (Phelps & Gazzaniga, 1992). Whereas non-human animals do not, humans find patterns in sequences of events, even when told the sequences are random (Wolford et al., 2000). If language encodes meaningful relations at different levels in the language system, pattern-matching skills allow humans to extract those patterns that are encoded in language. Those skills would consequently strengthen our language skills, which would strengthen our pattern-matching skills (Christiansen & Chater, 2008; Deacon, 1998; Louwerse, 2021).

Using the formalized model of information theory that quantifies information, the current study demonstrates that straightforward language statistical cues reduce entropy considerably, with word embedding propagating such biases throughout the language system. These findings suggest that language-internal statistical structure can support the extraction and propagation of meaning once at least some linguistic symbols have been grounded in external information. Moreover, the findings demonstrate how minimal grounding of words to their referents facilitates meaning extraction through language statistics. These findings give rise to reconsidering some important assumptions and subsequent theories in the language and cognitive sciences.

## Data Availability

The data used in this paper are available at: https://static-content.springer.com/esm/art%3A10.3758%2Fs13428-012-0314-x/MediaObjects/13428_2012_314_MOESM1_ESM.zip and https://osf.io/pv2dh.

## Code availability

All code are available at: https://osf.io/pv2dh.
